# Posttraumatic ascending myelopathy after spinal cord injury in a young man: A case report

**DOI:** 10.1002/ccr3.5997

**Published:** 2022-08-05

**Authors:** Mostafa Esmaeilnia, Mona Asadi, Hussein Sharara

**Affiliations:** ^1^ Department of Neurological Surgery, Imam Khomeini Hospital Complex Tehran University of Medical Sciences Tehran Iran; ^2^ Neurologist, Department of neurology Sabzevar University of Medical Sciences Sabzevar Iran; ^3^ Resident of neurology Mashhad University of Medical Sciences Mashhad Iran

**Keywords:** neurologic deterioration, rare condition, spinal cord, vertebrae fracture

## Abstract

A 29‐year‐old man developed subacute posttraumatic ascending myelopathy 5 days after a spinal cord injury. He developed a fever and a blood culture showed an Alkaligenes spp. infection. Despite antibiotic and high‐dose corticosteroid therapy, same neurological deficits persisted, and a follow‐up MRI showed atrophy and swelling in the cervical cord.

## Graphical abstract

Subacute posttraumatic ascending myelopathy (SPAM) is an extremely rare condition, mainly happens in male patients with traumatic spinal cord injury. SPAM has a poor prognosis and no effective treatment for this neurological deterioration.

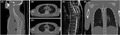

## 1 INTRODUCTION

Spinal cord injury (SCI) should be named as a disaster for the patient, family, and society. SCI results in degrees of paralysis, sensory loss, and bowel and bladder dysfunction.^1^ This condition not only poses a heavy psychological and physical burden on the patient, but also causes problems for the society and families. This condition may be traumatic or non‐traumatic. The main cause of traumatic cases is road accident and the main causes of non‐traumatic cases are tumors and degenerative disorders of the spine.^2^

A varying incidence for SCI is reported. The incidence of this condition in developed countries is estimated as 13.0–163.4 per million people in developed countries. This data is reported to be in a range of 13.0–220.0 per million people in non‐developed countries. The prevalence of this condition is also reported to be in a range of 440.0–526.0 per million general population.^1^ Moreover, the prevalence of SCI in Iran is estimated to be 90.0 per million people.^3^

Besides the above‐mentioned problems and burdens, there is no curative treatment for SCI and the treatment is usually supportive. Moreover, different neurological deterioration may happen irrelevant to the primary spine instability. The acute processes mainly affect the adjacent segments usually due to the vascular disorders. However, subacute changes may also happen within days or weeks.^4^ These include conditions like syringomyelia or subacute posttraumatic ascending myelopathy (SPAM).^5^ In fact, delayed ascending myelopathy is a rare condition that is reported in some previous reports.^4^, ^5^, ^6^, ^7^ The exact pathophysiology of SPAM is not fully understood and the report of different cases may help to the investigations with this regard.^7^ Here, we report a case of SPAM in a 29‐year‐old man, which was developed around a week after the injury.

## 2 CASE PRESENTATION

**FIGURE 1:**
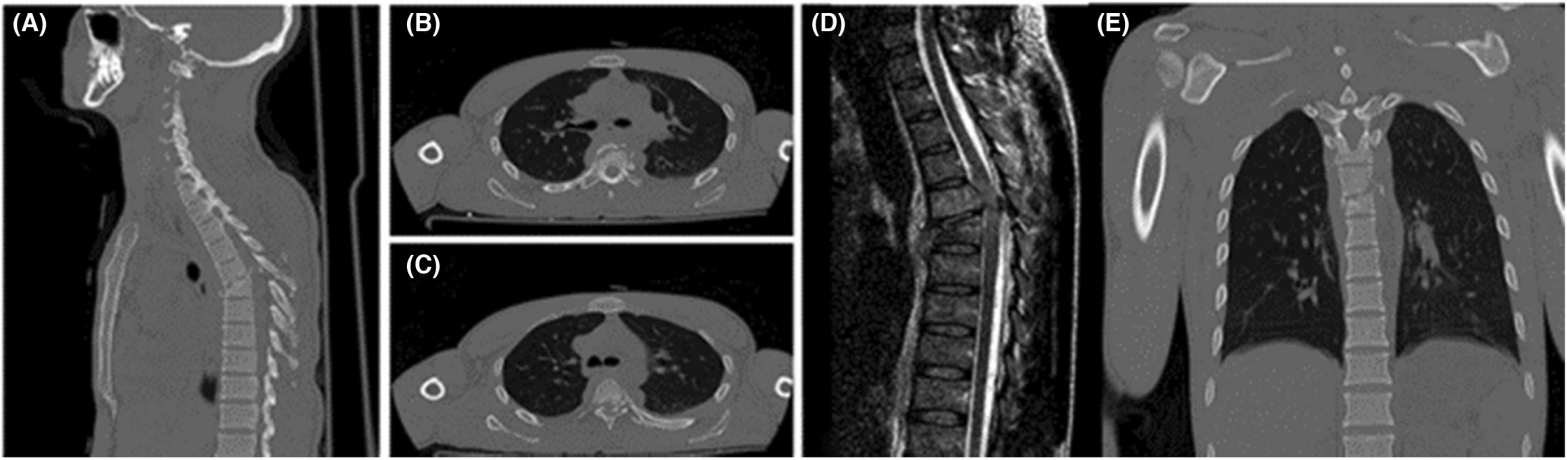
Preoperative computerized tomography (CT) scan and MRI of the thoracic spine show T5 & T6 fractures with kyphotic deformity. (A,B,C,E) Sagittal and Axial CT reconstruction (bone window) of the thoracic spine. (D) MRI sagittal view of the thoracic T2‐weighted sequence shows prominent cord compression

A 29‐year‐old (75 kg) male was transported to our emergency department after a slump block fell on him when he was working in a well at a building under construction. At initial assessment, he was conscious but hypotensive and both legs were paralyzed and he had urinary incontinence. He complained about extreme pain at upper posterior aspect of his chest. After volume resuscitation, cervical and thoracic spine computed tomography (CT) scan was performed. Axial images at 0.8 mm intervals with sagittal reformation revealed T5/T6 kyphotic deformity with impaction fractures of the superior endplate of T6 and inferior endplate of T5, which was defined as T5/T6 type C fracture according to the AO classification (Figure 1A,B,C,E). Posterior bony elements also had been injured. The cervical spine was intact and he had no neck pain.

In neurological examination the patient was complete paraplegic (Frankel scale A) and areflexic. Sensory level was T5 (a little above the level of xiphoid process) and involvement of the bowel and bladder was apparent. Motor and sensory function of the arms was normal. He was treated with intravenous methylprednisolone 30 mg/kg bolus followed by 5.4 mg/kg/h for 23 h (NASCIS II protocol). The next day, Magnetic resonance imaging (MRI) was performed and then patient transferred to the operating room. MRI revealed significant cord compression and high signal in T2 weighted images at the T5/T6 level (Figure 1D).

During surgery, T5 and T6 laminectomy was performed to decompress the spinal cord and pedicular screw and rod in T3 to T8 vertebras was inserted to fix the spinal column and posterolateral fusion was done. Minor CSF leak was seen after laminectomy that was probably due to tearing of the ventral aspect of the thecal sac but the dorsal surface was intact. After operation the general condition of the patient was good, but he complained of pain in the neck and scapula, that was lasted 4 days. On day 5, he had fever (T = 38.5°C) for 24 h. Empirical antibiotic therapy initiated for 3 days. The surgical wound was good without any complication and on day 8 patient was discharged from hospital. After 2 days (postinjury day 11), patient returned to the hospital with chief complaint of arm weakness and fever. On examination, in upper extremities muscle strength was 4/5 at proximal muscles and 2/5 in distal (hand grip) and sensory perception was decreased prominently. He was complete paraplegic likewise. Sepsis workup performed and blood culture became positive (after more than 48 h) with Alkaligenes spp. and intravenous imipenem 500 mg QID was started and continued for 2 weeks. Moreover, the patient underwent lumbar puncture and cerebrospinal fluid (CSF) analysis showed no evidence of meningitis. The glucose level of CSF was 58 mg/dl, the protein was 950 mg/dl, the white blood cell, and red blood cell count were negative. The Lung CT scan and blood tests including complete blood cell count, INR, PT, PTT, C‐reactive protein and erythrocyte sedimentation rate were normal.

**FIGURE 2:**
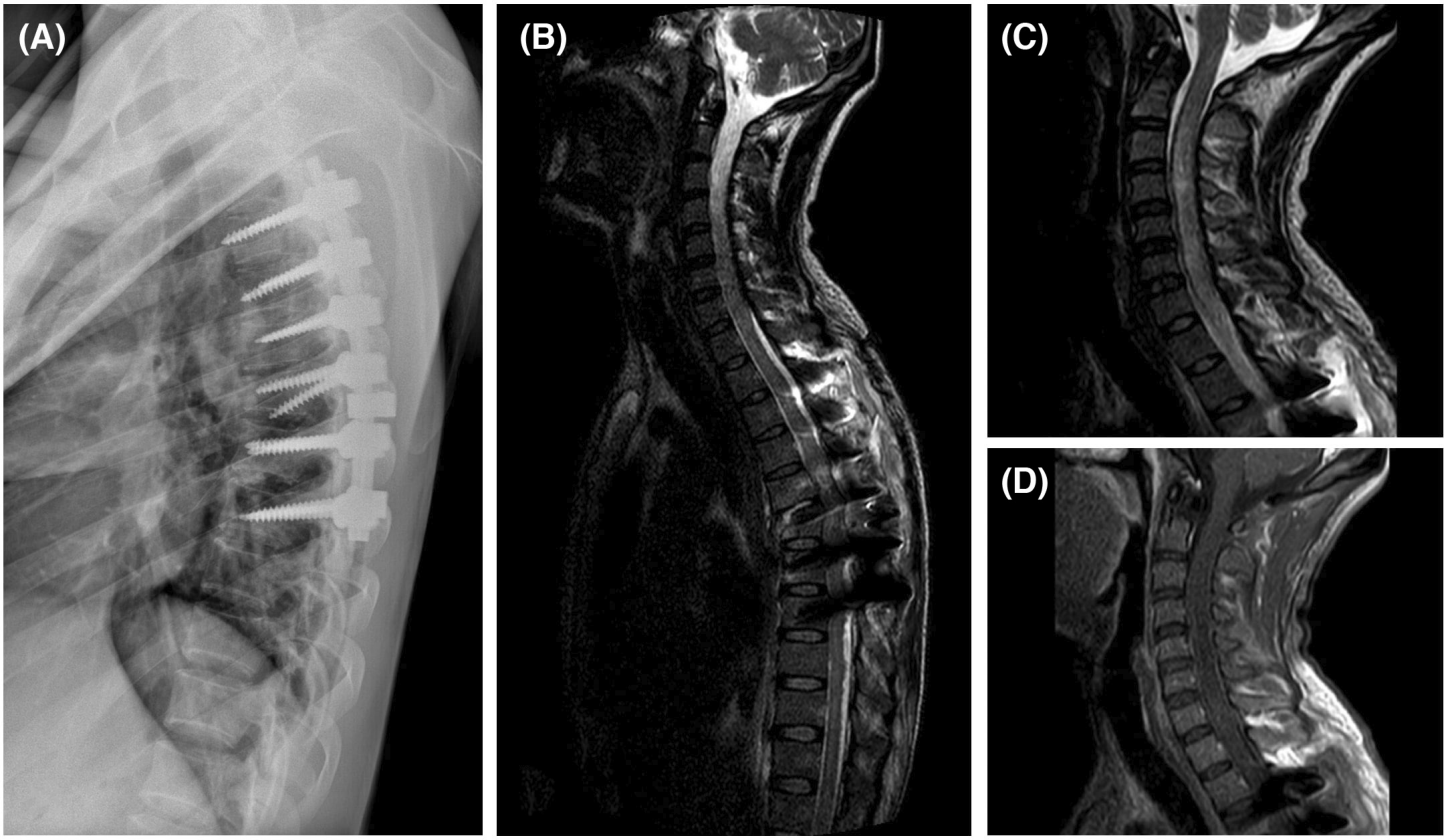
(A) Postoperative plain radiography of the thoracic spine in a lateral view shows Posterior long segment fixation by pedicle screw. (B and C) Sagittal reconstruction T2 weighted images of cervical and thoracic spine MRI on postinjury day 14 show increased signal and swelling between C3 and C7. Artifact from spinal instrumentation obscured visualization of cord segments in midthoracic region (D)

**FIGURE 3:**
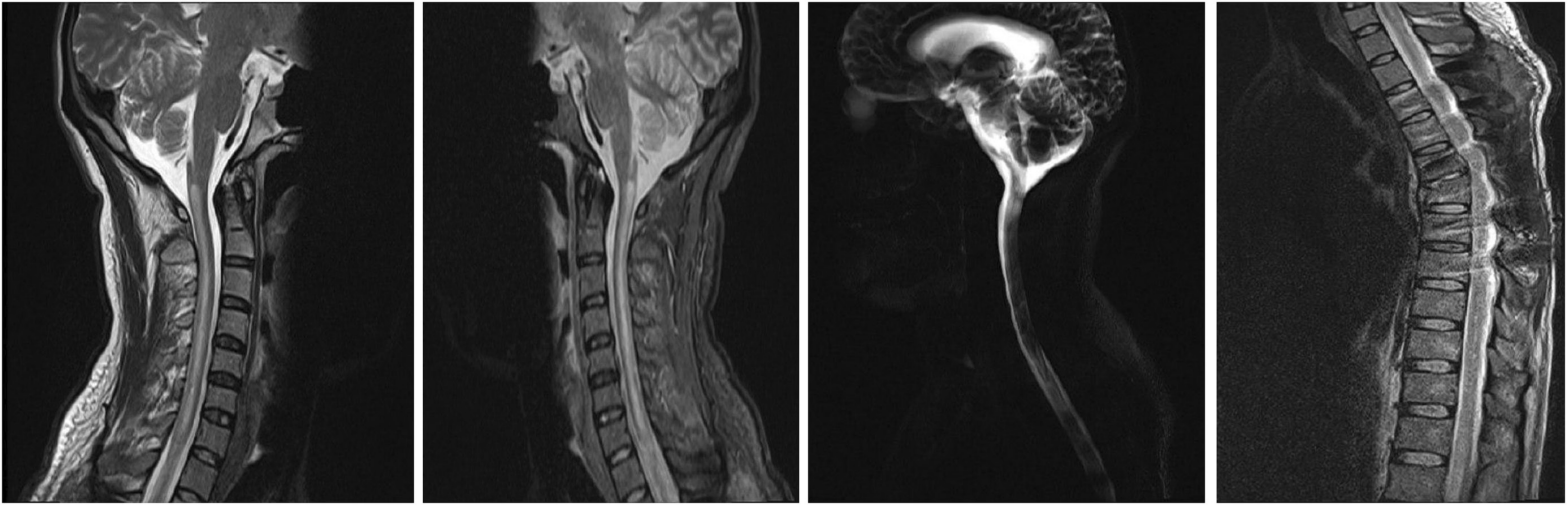
Follow‐up MRI of the patient in cervical and thoracic sequences after 50 days

The T1‐weighted sequence with gadolinium shows heterogeneous intramedullary signal without any enhancement. A new magnetic resonance imaging (MRI) study performed on postinjury day 14. The cervical and thoracic spine showed increased T2 weighted signal and cord swelling from C3 to C7 (Figure 2B,C). High‐dose methylprednisolone (500 mg daily) was initiated and slowly tapered in 3 weeks. After 8 weeks the patient still has the same neurological deficits. In follow‐up MRI, cervical cord appears slightly atrophic with significant reduction in swelling and signal. The follow up imaging after 50 days is demonstrated in Figure 3. Upper extremity forces were 4 out of 5 after this time interval.

## 3 DISCUSSION

The first case of SPAM was reported by Frankel in 1969 in a 40 years old man with T12 fracture with paraplegia. The lesion showed a 38.9 centigrade degrees' fever and an ascending pattern to the C7 level in the patient, within 6 to 15 days after the injury.^8^ However, this type of neurological deterioration was not noticed by other spine and neurosurgeons for 30 years, until 1999 when Aito et al. reported another similar case.^9^ The disorder is defined as neurological deterioration ascending four or more spinal segments above the injured site, within the first few weeks after SCI. Moreover, more than 90% of the cases are young or middle‐aged. Based on the previous reports, the incidence of SPAM varies between 0.42 to about 1% of SCI cases.^10^, ^11^ Furthermore, men are more prone to this condition than women, with a male to female ratio of 5 to 1. The injury level is reported to be at the thoracic level in 33.3% of the SPAM cases.^11^ The case of our study was also a young man with thoracic injury level that ascended to the cervical segments.

The exact mechanism of this disease is still under question. Several underlying pathophysiologic pathways have been issued with this regard. The elevation of CSF pressure due to occlusion in case of debris and hemorrhage is one of these proposed causes. Arterial and venous occlusions are the other causes. As the level of injuries in most SPAM cases is at the thoracic level, the occlusion of great artery of Adamkiewicz. However, the theory is not supported by angiographies of the region. Moreover, arterial occlusion results in an acute condition and not a subacute situation. Another proposed hypothesis that is supported by the results of autopsies is congestive ischemia due to the venous thrombosis.^11^, ^12^ However, there is a high rate of thrombosis in SCI cases and still SPAM happens in few cases. This relatively makes the hypothesis under the question. Hypotension is also believed to be another cause of venous stasis and occlusion. Still, this hypothesis does not support the progressive manner of this disease. Due to the accompanying fever in many SPAM cases, infection is another proposed probable mechanism. However, the CSF findings and autopsies do not confirm this, too. Moreover, the patients respond effectively to the corticosteroid therapy that further denies this hypothesis. Inflammatory and autoimmune responses are also accused, but the involvement does not exceed to other parts of CNS than spinal cord. It seems the mechanism of this condition have still remained under question and further investigations are needed. Maybe the condition is multifactorial and a combination of the above mentioned may cause the involvement.^8^, ^13^ The case of our study also had a fever with the culture of Alkaligenes spp from the blood sample.

Although SPAM is a rare neurological deterioration, it is well recognized according to typical clinical manifestation and MRI characteristics. History and physical examination are an important part with this regard. Typical characteristics of SPAM are that the patients suffered from neurological deterioration after a few days or weeks of latent clinical stability.^14^ Some patients complained of pain in the arms, shoulder, scapula, neck, chest or trunk before the appearance of motor weakness. The golden standard modality in diagnosing SPASM is MRI. MRI usually demonstrates a central area of high‐intensity signal on T2‐weighted sequences that ascended at least four segments cephalad to the initially injured. Lumbar puncture usually shows block in CSF flow below the injured site. Moreover, CSF assessment usually shows high protein level and neutrophils. The culture is also negative in these patients.^11^, ^13^ The condition can even present itself in a subacute way, as Belanger et al. presented three delayed cases that presented their self 1 to 2 weeks after spinal cord injury.^15^

Kovanda et al.^16^ also reported another case in a 15‐years‐old boy. The patient presented with paraplegia and loss of the sensation at the level of T4, after a motor bike accident. He developed bilateral upper extremity weakness, 8 days after the injury. This condition was progressive for around 40 days and finally the patient was diagnosed as SPAM.

The treatment can be a combination of supportive treatment, medications and drug administration, and even surgery. Supportive treatment is conducted by monitoring vital signs and blood pressure, prevention hypotension, oxygen therapy, and ventilation in some cases. The medication therapy is reported to be mainly by administration of corticosteroids, anticoagulants, and broad‐spectrum antibiotics.^14^ Our case of study also received high dose of methylprednisolone and antibiotics. Operative management is also believed to reduce CSF pressure and may be helpful with this regard; however, further investigations are needed.^17^

The prognosis of SPAM is nearly poor. It is reported that many cases need prolonged hospitalization and even mechanical ventilation. Moreover, around 1 out of every ten SPAM cases die due to this condition. However, the prognosis is associated with the cord level of ascended myelopathy. In fact, the regression of the myelopathy to the injury level is extremely rare. Even after extensive and long treatment, a slight improvement of ≥1 level (s) is experienced by some patients.^11^

## 4 CONCLUSION

In conclusion, SPAM is an extremely rare condition, mainly happens in male patients with traumatic SCI, in a subacute manner. The underlying pathology of the disease should be further investigated. The medical treatment is based on the steroids, antibiotic, anticoagulants, and supportive cares. Surgery may also play a role; however, more studies are needed in case of this issue Patients' prognosis is poor and even the condition may lead to mortality. Further investigations are needed for a thorough conclusion about SPAM.

## AUTHOR CONTRIBUTIONS

All authors contributed to the study's conception and design. ME, and HS performed material preparation, data collection, and acquisition. MA, and HS involved in writing the first draft of the manuscript. All authors approved the final manuscript as submitted and agree to be accountable for all aspects of the work.

## CONFLICT OF INTEREST

The authors declare that there are no conflicts of interest.

## CONSENT

This study was approved by the Ethics Committee of Tehran University of Medical Sciences, Tehran, Iran. All tests were carried out in compliance with the institution's specified rules and regulations. Furthermore, the patient's written informed consent was acquired for the publishing of this case report.

## Data Availability

The data are also available on request.
